# Joint effects of prenatal exposure to per- and poly-fluoroalkyl substances and psychosocial stressors on corticotropin-releasing hormone during pregnancy

**DOI:** 10.1038/s41370-021-00322-8

**Published:** 2021-04-06

**Authors:** Stephanie M. Eick, Dana E. Goin, Lara Cushing, Erin DeMicco, Sabrina Smith, June-Soo Park, Amy M. Padula, Tracey J. Woodruff, Rachel Morello-Frosch

**Affiliations:** 1 Program on Reproductive Health and the Environment, Department of Obstetrics, Gynecology and Reproductive Sciences, University of California, San Francisco, San Francisco, CA USA; 2 Department of Environmental Health Sciences, Fielding School of Public Health, University of California, Los Angeles, Los Angeles, CA USA; 3 Environmental Chemistry Laboratory, Department of Toxic Substances Control, California Environmental Protection Agency, Berkeley, CA USA; 4 Department of Environmental Science, Policy and Management and School of Public Health, University of California, Berkeley, Berkeley, CA USA

**Keywords:** Per- and poly-fluoroalkyl substances, Stress, Pregnancy, Health disparities

## Abstract

**Background:**

Prenatal exposure to per- and poly-fluoroalkyl substances (PFAS) and psychosocial stressors has been associated with adverse pregnancy outcomes, including preterm birth. Previous studies have suggested that joint exposure to environmental chemical and social stressors may be contributing to disparities observed in preterm birth. Elevated corticotropin-releasing hormone (CRH) during mid-gestation may represent one biologic mechanism linking chemical and nonchemical stress exposures to preterm birth.

**Methods:**

Using data from a prospective birth cohort (*N* = 497), we examined the cross-sectional associations between five individual PFAS (ng/mL; PFNA, PFOA, PFOS, PFHxS, and Me-PFOSA-AcOH) and CRH (pg/mL) using linear regression. PFAS and CRH were measured during the second trimester in serum and plasma, respectively. Coefficients were standardized to reflect change in CRH associated with an interquartile range (IQR) increase in natural log-transformed PFAS. We additionally examined if the relationship between PFAS and CRH was modified by psychosocial stress using stratified models. Self-reported depression, stressful life events, perceived stress, food insecurity, and financial strain were assessed using validated questionnaires during the second trimester and included as binary indicators of psychosocial stress.

**Results:**

An IQR increase in PFNA was associated with elevated CRH (*β* = 5.17, 95% confidence interval [CI] = 1.79, 8.55). Increased concentrations of PFOA were also moderately associated with CRH (*β* = 3.62, 95% CI = −0.42, 7.66). The relationship between PFNA and CRH was stronger among women who experienced stressful life events, depression, food insecurity, and financial strain compared to women who did not experience these stressors.

**Conclusions:**

This cross-sectional study is the first to examine the relationship between PFAS exposure and CRH levels in mid-gestation. We found that these associations were stronger among women who experienced stress, which aligns with previous findings that chemical and nonchemical stressor exposures can have joint effects on health outcomes.

## Introduction

Exposure to per- and poly-fluoroalkyl substances (PFAS) is ubiquitous in the US, with >95% of individuals having detectable serum levels [1]. PFAS are endocrine disrupting chemicals (EDCs) that are highly persistent in the environment and prevalent in a wide range of consumer products including cookware, clothing, and food packaging, as well as food and drinking water sources [2–4]. More than 4000 PFAS have been identified and levels of some PFAS bioaccumulate in humans [2]. Previous studies have linked prenatal PFAS exposure to increased risk of adverse pregnancy outcomes. For example, increased concentrations of perfluorooctanoic acid (PFOA), perflucorooctane sulfonic acid (PFOS), and perfluorononanoic acid (PFNA) have been associated with increased odds of preterm birth (defined as gestational age at birth <37 weeks) and a reduction in gestational age at birth [5, 6]. Other studies have shown that PFAS chemicals are easily transmitted to the developing fetus via the placenta [7] and present in the cord blood of newborns [8]. Despite the consistency in these associations, the biologic pathways that link PFAS to preterm birth largely remain unknown.

Experiences of psychosocial stressors during pregnancy, including stressful life events, poor neighborhood quality, and responses to such stressors, such as depression and anxiety, have also been associated with preterm birth [9–12]. Furthermore, there may be a cumulative or joint effect of chemical and psychosocial stressors [13]. This may be especially relevant for lower socioeconomic status (SES) individuals, who are often exposed to multiple environmental and social stressors simultaneously [14]. It is possible that these chemical and nonchemical stressors affect similar biologic mechanisms and thus may lead to adverse outcomes through similar pathways.

The hypothalamic pituitary adrenal (HPA) axis has emerged as one mechanism linking prenatal stress exposure to preterm birth [15]. It is hypothesized that experiencing psychosocial stress activates the maternal HPA axis, increasing cortisol production, which is followed by production of corticotropin-releasing hormone (CRH). Since the placenta also produces CRH and CRH levels during pregnancy are considered part of a “biological clock” regulating the length of gestation [16, 17], early excess CRH production may result in preterm birth [15]. This pathway has been examined in animal models [18] and human studies have shown that certain psychosocial stressors are associated with elevated CRH during the second and third trimesters of pregnancy [19–22].

To date, no studies have examined the association between prenatal PFAS exposure and CRH during pregnancy. However, increasing concentrations of other classes of EDCs, including phenols, parabens, and phthalates, have been associated with alternations in HPA axis function in animal models [23] and during human pregnancy [24]. For example, increases in CRH levels between 24 and 28 weeks gestation have been observed in association with increasing 2,4-dichlorophenol and triclosan levels [25]. In contrast, inverse associations between phthalate metabolites and CRH, measured at 16–20 and 24–28 weeks gestation, were detected in a pregnancy cohort in Puerto Rico [26]. Elevated levels of phthalates and phenols have also been linked to an increased risk of preterm birth [27, 28]. Previous work has identified a joint effect of stressful life events and EDCs with respect to gestational length at delivery. In these studies, inverse associations between certain phenol and phthalate metabolites and gestational age were stronger among women who experienced stressful events [29, 30].

In the present study, we utilized data from a demographically diverse group of pregnant women in San Francisco, CA to assess the relationship between PFAS exposures and CRH during pregnancy. We hypothesized that increasing PFAS exposure would be associated with elevated CRH during the second trimester and that these associations would be stronger among women who experienced psychosocial stress.

## Methods

### Study population

This study utilized data from 497 participants enrolled in the Chemicals in Our Bodies (CIOB) cohort, which has been previously described in detail [31]. Briefly, CIOB was designed to examine the cumulative effects of environmental chemicals and psychosocial stressors on fetal growth and offspring development. Women were recruited during their second trimester from three University of California, San Francisco hospitals between 2014 and 2018. Participants recruited from the Zuckerberg San Francisco General Hospital were primarily lower income and enrolled in Medi-Cal (California’s Medicaid program), whereas women recruited from Moffitt Long and Mission Bay Hospitals were economically diverse, and the majority had private health insurance. Women were eligible for enrollment in CIOB if they were ≥18 years of age, not pregnant with multiples, and spoke English or Spanish as their primary language. As part of the study, mothers consented to study staff accessing their medical records. The Institutional Review Boards at the University of California, San Francisco (10-00861) and Berkeley (2010-05-04) approved CIOB and all participants provided written, informed consent prior to participating. All study personnel were blinded to participants’ CRH and PFAS levels.

### Per- and poly-fluoroalkyl substances (PFAS)

Maternal serum samples were collected during the second trimester between 12 and 28 weeks gestation in red top tubes and stored at −80 °C until analysis for 12 PFAS. Analyses were performed by the Environmental Chemical Laboratory at the California Department of Toxic Substances Control. Method detection limits (MDL) were calculated as three times the standard deviation (SD) of the blank concentrations for all PFAS. PFAS were analyzed by injection onto an automated on-line solid phase extraction method coupled to liquid chromatography and tandem mass spectrometry. Additional details regarding PFAS measurement and quality assurance and quality control is provided elsewhere [8, 32]. We focused our analysis on those PFAS chemicals with >80% detection, which included PFNA, PFOS acid, PFOA, methyl-perfluorooctane sulfonamide acetic acid (Me-PFOSA-AcOH), and perfluorohexanesulphonic acid (PFHxS). This cut point was utilized for consistency with our prior work in this cohort [32]. While other PFAS compounds had machine-read values >80%, variability in PFAS levels across our population for these compounds was limited. In our analysis, measurements below the MDL were assigned the machine-read value if a signal was detected (<2% of observations). If no signal was detected, measurements below the MDL were treated as missing (<0.5% of observations). PFAS (ng/mL) concentrations were right-skewed and natural log-transformed for analysis.

### Corticotropin-releasing hormone (CRH)

Blood samples were collected during the second trimester (mean 20.7 weeks, range 12–28 weeks) and were centrifuged to extract plasma. Plasma samples were subsequently aliquoted and frozen at −80 °C until analysis. Analysis for CRH levels (pg/mL) was conducted by the Fisher Lab at the University of California, San Francisco using enzyme immunoassay kits (Phoenix Pharmaceutical, Burlingame, CA) and standard operating protocols. Samples were acidified and loaded onto pretreated SEP-COLUMNs, and resultant eluent was evaporated and redissolved in RIA buffer for radioimmunoassay. If subsequent concentrations were not within range of detection, samples were diluted or reconcentrated accordingly.

### Psychosocial stress

Psychosocial stressors and responses to stress were assessed via an interview questionnaire administered by study personnel during a second trimester prenatal care visit. Psychosocial stressors included stressful life events [33, 34], financial strain [35], and food insecurity [36, 37]. Perceived stress [38] and depression [39] were included as measures of stress response. A detailed description of the categorization of psychosocial stressors and stress response measures is available elsewhere [40] and is summarized below.

#### Stressful life events

Women were considered to have experienced stressful life events if they reported experiencing two or more of the following events within the last 12 months: a close family member was hospitalized, separation or divorce from her partner, she or her partner lost their job, she moved to a new address, a close family member experienced immigration problems, she argued with her partner more than usual, her partner did not want her to be pregnant, she was in a physical fight, she could not pay her bills, her partner had legal trouble, someone close to her was drinking or using drugs, or someone close to her passed away [33, 34].

#### Financial strain

Women were classified as experiencing financial strain if their annual household income was below the 2017 San Francisco county poverty line or they reported finding it difficult to pay for basic necessities, such as food, housing, medical care, or utilities [35].

#### Food insecurity

If women reported that within the past 12 months they had skipped meals, ate less than they should, or were hungry but did not eat because there was not enough money for food, they were classified as being food insecure. Women were also considered to be food insecure if they reported that the food they bought did not last or if there was not enough money for more food or if they could not afford to eat balanced meals [36, 37].

#### Perceived stress

Perceived stress was assessed using the Perceived Stress Scale-4 (PSS-4) [38, 41]. The range of scores on the PSS-4 was 0–13. For consistency with prior work, the standardized cut point of >9 was used to denote experiences of moderate or severe perceived stress [42, 43].

#### Depression

The 10-item Center for Epidemiologic Studies-Depression (CES-D) was used to measure depression [39]. CES-D scores ranged from 0 to 30, and the clinical cut point for depressive symptoms (≥16) was used to classify participants as having experienced depression [44].

### Demographics

Maternal age at enrollment, pre-pregnancy body mass index (BMI; kg/m^2^), parity, gestational age at study visit and gestational age at delivery were obtained from the mother’s abstracted medical record. On the medical record, gestational age is calculated using the clinician’s best estimation of chronological gestational age based on last menstrual period, early ultrasound, and/or in vitro fertilization date. Maternal race/ethnicity (Non-Hispanic (NH) White, NH-Black, NH-Asian/Pacific Islander, NH-Other/Multi-Racial and Latina), marital status (married, living with a partner, single), and maternal educational attainment (less than high school, high school or some college, college degree, graduate degree) were obtained via self-report at the second trimester interview.

### Statistical analysis

We examined the distribution of CRH and PFAS across demographic characteristics and psychosocial stressors using geometric means and geometric SD. Loess curves were used to assess the relationship between CRH and individual PFAS, and no evidence of nonlinearity was observed. Thus, linear regression models were used to estimate unadjusted and adjusted associations between PFAS, psychosocial stressors, and CRH. Coefficients were standardized to reflect the change in CRH associated with an interquartile range (IQR) increase in PFAS levels for ease of interpretation. An IQR increased was defined by the difference between the 75th and 25th percentiles. QQ plots were examined to ensure that regression residuals were normally distributed. We a priori controlled for gestational age at study visit in all models (unadjusted and adjusted), as CRH increases throughout pregnancy and we previously observed elevated PFAS and preterm birth odds ratios in this study population [32]. Maternal age, race/ethnicity, education, and parity were included as additional covariates in final adjusted models because they were associated with CRH, stress, and PFAS in bivariate analyses, and previous literature suggests an association with our exposures and outcome [45, 46]. We additionally adjusted for pre-pregnancy BMI and nativity as a sensitivity analysis and point estimates did not change substantially. A complete case analysis was used for all models.

We examined psychosocial stressors, including stressful life events, perceived stress, depression, food insecurity, and financial strain, as potential effect modifiers of the relationship between PFAS and CRH. In these analyses, we estimated the joint effects of PFAS and psychosocial stressors by examining the additive association between individual PFAS and CRH stratified by levels of each binary stressor, adjusting for gestational age at sample collection, maternal age, race/ethnicity, education, and parity. Separate models included *p* values for the interaction terms between PFAS and binary psychosocial stressors, which we used to assess additive interaction at *p* < 0.10. All statistical analyses were conducted in R Version 4.0.1.

## Results

The majority of women in the CIOB study population were >30 years of age (73%), married (66%), and had a college (23%) or graduate education (36%). More than half of women (57%) experienced at least two stressful life events within the past year and 34% experienced financial strain (Table 1). The geometric mean of CRH was higher among White women compared to Latina women (33.02 versus 25.28 pg/mL). Women who were classified as experiencing financial strain and depression also had elevated CRH levels relative to women who did not report these stressors (Table 1). Perceived stress, depression, financial strain, food insecurity, and stressful life events were moderately to strongly correlated with one another [40].

**Table 1:** Description of Chemicals in Our Bodies study population and geometric mean (geometric standard deviation) of corticotropin-releasing hormone (pg/mL) across demographic characteristics.

	*N* (%)	CRH geometric mean (geometric SD)
Maternal age, years		
18–24	54 (11 %)	28.19 (2.03)
25–29	66 (13 %)	29.93 (1.92)
30–34	172 (35 %)	29.5 (2.21)
≥35	191 (38 %)	31.41 (2.34)
Missing	14 (3%)	
Maternal education		
Less than high school	58 (12 %)	22.83 (2.16)
High school degree or some college	135 (27 %)	26.94 (2.06)
College degree	114 (23 %)	31.75 (2.17)
Graduate degree	181 (36 %)	33.79 (2.25)
Missing	9 (2%)	
Maternal race/ethnicity		
White	183 (37 %)	33.02 (2.16)
Black	36 (7 %)	34.66 (1.98)
Asian/Pacific Islander	82 (16 %)	32.07 (2.37)
Latina	172 (35 %)	25.28 (2.17)
Other/Multi-Racial	14 (3 %)	33.6 (1.93)
Missing	10 (2 %)	
Pre-pregnancy body mass index		
Underweight (<18.5 kg/m^2^)	12 (2 %)	18.45 (2.88)
Normal (18.5–24.9 kg/m^2^)	230 (46 %)	32.17 (2.12)
Overweight (25–29.9 kg/m^2^)	126 (25 %)	30.57 (2.35)
Obese (≥30 kg/m^2^)	88 (18 %)	25.3 (2.05)
Missing	41 (8.2%)	18.45 (2.88)
Parity		
No prior births	239 (48 %)	32.54 (2.04)
One or more prior births	251 (51 %)	28.4 (2.28)
Missing	7 (1 %)	
Marital status		
Married	326 (66 %)	31.67 (2.23)
Living together	104 (21 %)	25.61 (2.15)
Single	57 (11 %)	28.49 (1.97)
Missing	10 (2 %)	
Perceived stress		
Yes	62 (12 %)	29.57 (2.19)
No	422 (85 %)	31.53 (2.29)
Missing	13 (3%)	
Depression		
Yes	37 (7 %)	30.09 (2.22)
No	420 (85 %)	28.54 (2.05)
Missing	40 (8%)	
Stressful life events		
Yes	284 (57 %)	28.57 (2.29)
No	201 (40 %)	30.82 (2.13)
Missing	12 (2%)	
Financial strain		
Yes	170 (34 %)	32.32 (2.25)
No	262 (53 %)	28.37 (2.18)
Missing	65 (13%)	
Food insecurity		
Yes	76 (15 %)	30.69 (2.21)
No	409 (82 %)	27.4 (2.01)
Missing	6 (1%)	

*N* = 497.

*SD* standard deviation.

The five PFAS that were the focus of our analysis were detected in over 98% of women. The distribution of PFAS varied widely across compounds (Table 2). The median level was highest for PFOS (1.92 ng/mL) and PFOA (0.76 ng/mL), and lowest for Me-PFOSA-AcOH (0.05 ng/mL). The distribution of PFAS with <80% detection is shown in Table S1. The median level of CRH was 32.56 pg/mL.

**Table 2:** Distribution of second trimester plasma levels of corticotropin-releasing hormone (pg/mL) and second trimester serum levels of per- and poly-fluoroalkyl substances (ng/mL) (*N* = 497).

	% Above MDL	% Machine readable	Geometric mean (geometric SD)	Percentile				
				5th	25th	50th	75th	95th
CRH	100.00	100.00	30.10 (2.19)	8.62	17.77	32.56	50.73	99.21
PFNA	98.79	99.60	0.30 (1.92)	0.10	0.20	0.30	0.43	0.85
PFOA	99.80	100.00	0.73 (1.97)	0.25	0.46	0.76	1.12	2.11
PFHxS	100.00	100.00	0.36 (2.26)	0.11	0.20	0.32	0.58	1.49
PFOS	100.00	100.00	1.86 (2.07)	0.51	1.18	1.92	3.11	6.00
Me-PFOSA-AcOH	98.79	99.80	0.05 (2.07)	0.02	0.03	0.05	0.08	0.18

*SD* standard deviation; *MDL* method detection limit.

In our study population, PFAS levels were generally higher among women who were older, married, and had at least a college education (Table S2). With the exception of PFOS, levels of all PFAS were lower among Latina and Black women relative to other racial and ethnic groups. Levels of PFNA, PFOA, and PFOS were lower among women who experienced food insecurity and financial strain (Table S2).

**Fig. 1:**
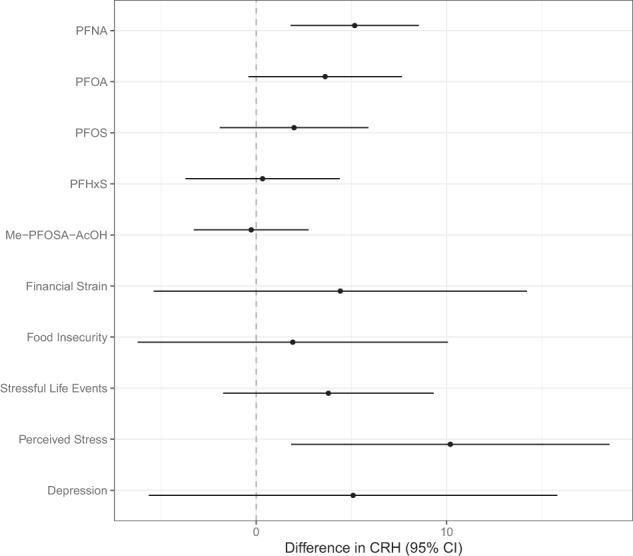
Adjusted linear regression coefficients and 95% confidence intervals for second trimester CRH concentrations (pg/mL) in maternal plasma in relation to psychosocial stressors and an interquartile range increase in second trimester PFAS concentrations (ng/mL) in maternal serum. PFAS are natural log-transformed. Models adjusted for gestational age at study visit, maternal age (continuous), maternal education, maternal race/ethnicity, and parity.

In unadjusted models adjusted only for gestational age at visit, we observed an increase in CRH (pg/mL) per unit IQR increase in PFNA (*β* = 6.26, 95% confidence interval [CI] = 3.31, 9.21), PFOA (*β* = 5.11, 95% CI = 1.70, 8.52), and PFOS (*β* = 4.38, 95% CI = 1.06, 7.70). Only the PFNA effect remained relatively unchanged after adjustment for the additional covariates (*β* = 5.17, 95% CI = 1.79, 8.55) (Fig. 1 and Table S3). In adjusted models, a high level of perceived stress was associated with higher CRH in pg/mL compared to women who did not experience perceived stress (*β* = 10.2, 95% CI = 1.82, 18.57). Depression, stressful life events, food insecurity, and financial strain were associated with CRH in the direction hypothesized after adjustment, however the confidence intervals included the null value (Fig. 1 and Table S4).

**Fig. 2:**
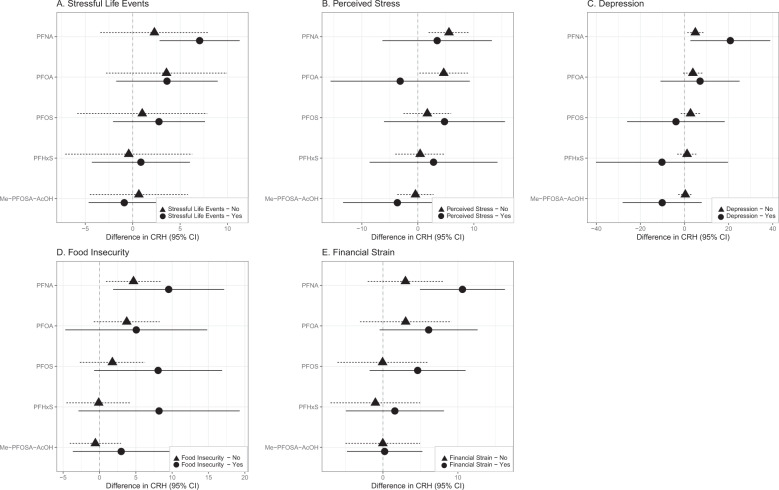
Adjusted linear regression coefficients and 95% confidence intervals for second trimester CRH concentrations (pg/mL) in maternal plasma with an interquartile range increase in second trimester PFAS concentrations (ng/mL) in maternal serum stratified by binary indicators of psychosocial stress. PFAS are natural log-transformed. Models adjusted for gestational age at study visit, maternal age (continuous), maternal education, maternal race/ethnicity, and parity.

In adjusted linear regression models stratified by each stressor, we found that the relationship between PFNA and CRH was stronger among women who experienced stressful life events (*β* = 7.06, 95% CI = 2.82, 11.3), depression (*β* = 20.77, 95% CI = 1.56, 39.99), food insecurity (*β* = 9.52, 95% CI = 1.72, 17.32), and financial strain (*β* = 10.61, 95% CI = 4.90, 16.33) compared to women who did not experience these stressors (Fig. 2 and Tables S5, S7–S9). Among women who did not experience perceived stress, an IQR increase in PFOA was associated with a 4.61 pg/mL increase in CRH (95% CI = 0.28, 8.95) (Fig. 2 and Table S6). Associations between PFOS, PFHxS, and CRH were stronger among women who were food insecure relative to those who did not report food insecurity (Fig. 2 and Table S8). In contrast, a modest and imprecise inverse association was observed between PFOS (*β* = −3.88, 95% CI = −27.33, 19.56), PFHxS (*β* = −10.23, 95% CI = −41.98, 21.52), Me-PFOSA-AcOH (*β* = −10.12, 95% CI = −29.15, 8.91), and CRH among women who experienced depression. With the exception of PFNA, the relationship between PFAS and CRH among those who did not experience depression was null (Fig. 2 and Table S7). Tests for potentially interactive effects for each stressor and stress response were not significant at *p* < 0.1.

## Discussion

Among a diverse population of pregnant women in San Francisco, we found that an IQR increase in levels of PFNA in maternal serum was associated with higher CRH concentrations during the second trimester. The relationship between PFNA and CRH was stronger among women who experienced stressful life events, depression, food insecurity, and financial strain relative to women who did not experience these stressors but tests for interactive effects were not statistically significant. We also observed an association between perceived stress and higher prenatal CRH. Our findings contribute to the growing body of literature supporting a joint effect of environmental chemical and psychosocial stressors on biomarkers of stress response.

In our study population, we found that women who reported high levels of perceived stress had elevated levels of CRH compared to women who experienced no or low perceived stress when assessed at the second trimester. This finding is consistent with previous research. Among 85 pregnant women in Utah, the mean score on the PSS was marginally elevated among women with CRH ≥ 15 pcg/mL compared to those with CRH < 15 pcg/mL (21.1 versus 18.2) when CRH was measured between 14 and 20 weeks gestation [20]. Similarly, a path analysis conducted among Hispanic pregnant women in Texas found that perceived stress was positively associated with CRH at 22–25 weeks gestation [47]. In contrast, in our study population, none of the other stressors examined were associated with second trimester CRH. While these null associations are in contrast to our hypotheses and prior work [21, 22], they are consistent with prior findings. For example, among a prospective birth cohort in North Carolina, mid-pregnancy placental CRH (measured twice between 20 and 24–29 weeks gestation) was not elevated in response to prenatal depression and furthermore that study found no association between prenatal CRH and postnatal depression [48]. It is possible that we did not observe stronger effects between stress and CRH due to the timing of its measurement during pregnancy. We measured CRH during the second trimester (mean 20 weeks) and other studies have shown an association between stress and CRH generally measured CRH during the third trimester [19, 21, 22]. This may indicate that the third trimester is a more susceptible time period for psychosocial stress exposure. It is possible that CRH is also sensitive to more acute stress exposure. In our study population, we observed a positive association between CRH and perceived stress using the PSS, which asks about experiences of stress within the last month. Chronic stress exposure suppresses the HPA axis [15], which may decrease the release of CRH. This may explain why measures of chronic stress, including food insecurity, depression, financial strain, and stressful life events, were not independently associated with CRH in our study.

PFAS bioaccumulate across the lifespan and exposure is ubiquitous among pregnant women in the US [2]. However, PFAS levels were lower in our cohort relative to other study populations, including NHANES [31], which could be due to some PFAS levels declining over time as a result of voluntary industry phase-outs of PFOA and PFOS. We also observed that PFAS levels were higher among those with higher educational attainment, which could be due to SES-related differences in product use and consumption of certain foods known to contain PFAS, including fish. PFAS are also excreted via breastmilk [2], which may explain why PFAS levels were lower among multiparous women in our study. These factors may be driving differences we observed in adjusted versus unadjusted models. Latina participants in our cohort also had lower PFAS levels relative to other racial and ethnic groups, which could be attributed to the high percentage of Latinas in our study born outside of the US and is consistent with past work [49].

Our study is the first to examine CRH as a potential physiologic response to PFAS exposure during pregnancy. Higher levels of CRH may represent one possible biologic pathway linking PFAS and stress exposures to preterm birth, shortened gestational length, fetal growth restriction, and early childhood neurodevelopment [50, 51]. Prior work has shown that levels of PFOA and PFOS decrease moderately from early pregnancy to birth [52], possibly as a result of transplacental transfer of PFAS. CRH, measured longitudinally, increases over the course of pregnancy and elevated CRH during early gestation (16–20 weeks) is associated with a marked increase in the risk of preterm birth [15]. Previously in the CIOB cohort, compared to women in the lowest tertile of PFNA exposure, women in the upper two tertiles had 1.88 (95% CI = 0.83, 4.30) and 2.06 (95% CI = 0.72, 5.89), respectively, increased odds of preterm birth [32]. While this finding was not statistically significant, the magnitude and direction of these effects suggest a potential dose–response relationship. Of all PFAS examined in relation to preterm birth in our previous work, PFNA had one of the strongest effects [32], which is consistent with our observations in this study with respect to PFNA and CRH levels.

Our prior work in the CIOB cohort has also shown that many of the stressors examined here have a joint effect on infant birthweight for gestational age [53]. CRH plays a critical role in regulating the physiologic stress response. Under the experiences of acute stress, the HPA axis is activated, leading to the release of adrenocorticotropic hormone, which signals the release of cortisol [15]. High cortisol levels signal to the hypothalamus to release CRH. Chronic activation of the HPA axis may lead to dysregulation of the neuroimmunological and neurohormonal systems, and may result in excess CRH and cortisol in maternal circulation [15]. This pathway may also be altered by endocrine disruption, as previous work has shown that pregnant women with high mercury and stress levels have a blunted morning cortisol response relative to women with lower mercury levels [54]. Elevated prenatal CRH levels have also been observed following exposure to some phthalate and phenol metabolites [25, 26]. The upstream biomarkers of physiologic effect associated with environmental chemical and social stressors deserve further exploration in future studies.

Our study has many important strengths. We included multiple measures of stress and responses to stress and our study population included a demographically diverse group of pregnant women. We also acknowledge our limitations. Our study population was not a random sample and thus findings may not be generalizable to other pregnant populations. PFAS, stress, and CRH were measured at the same time point, which raises temporality challenges typical of cross-sectional study designs. However, it is unlikely that CRH or stress would affect PFAS levels. Furthermore, our sample size was modest and this imprecision is reflected in the wide confidence intervals observed for some of our point estimates. In addition, we dichotomized all psychosocial stress measures in our analyses. While we did use previously establish cut points for all scales, this could have resulted in a loss of statistical power. Our stressful life events scale also did not capture the subjective assessment of stressful events, which may introduce misclassification depending on the perception of these events. Further, we did not make adjustments for multiple comparisons. Although adjustment for multiple comparisons is not always necessary [55] and we focused consistent trends rather than *p* values in interpreting our results, this may increase the possibility of chance findings. While an important strength of our study is that we examined the joint effects of chemical and nonchemical stressors, we did not examine the cumulative effects of these exposures. Last, cortisol levels and experiencing traumatic events during early pregnancy have been linked to increased risk of early fetal loss [56, 57] and as a result, our study findings may be subject to live birth bias [58].

## Conclusions

Our cross-sectional study is the first to examine the effects of PFAS exposure on CRH during pregnancy and our findings highlight a stress response biomarker of potential relevance for adverse pregnancy outcomes including preterm birth. We found modestly stronger associations among women who experienced maternal stress. Future studies should focus on the potential modifying effect of stress and its potential to increase susceptibility to the effects of environmental chemicals among pregnant women.

## Supplementary information

Supplemental tables
