# Supplementary material for: Joint effects of prenatal exposure to per- and poly-fluoroalkyl substances and psychosocial stressors on corticotropin-releasing hormone during pregnancy
Source: J Expo Sci Environ Epidemiol. 2021 Apr 6;32(1):27–36. doi: 10.1038/s41370-021-00322-8 (PMC8492777; doi:10.1038/s41370-021-00322-8)
Supplement: Supplementary file 1 — Supplemental tables [file 41370_2021_322_MOESM1_ESM.docx]

**Table S1. Distribution of second trimester plasma levels of corticotropin releasing hormone (pg/mL) and second trimester serum levels of per- and poly-fluoroalkyl substances (ng/mL) with <80% detection (N=497).**

|  | % Above MDL | % Machine Readable | Geometric Mean (Geometric SD) | Percentile | | | | |
| --- | --- | --- | --- | --- | --- | --- | --- | --- |
|  |  |  |  | 5^th^ | 25^th^ | 50^th^ | 75^th^ | 95^th^ |
| PFDeA | 69.22 | 92.56 | 0.18 (1.75) | <MDL | <MDL | 0.16 | 0.24 | 0.49 |
| PFUdA | 72.03 | 95.77 | 0.14 (1.84) | <MDL | <MDL | 0.14 | 0.23 | 0.40 |
| PFOSA | 2.41 | 45.88 | 0.03 (1.66) | <MDL | <MDL | <MDL | <MDL | <MDL |
| PFBS | 0.80 | 55.13 | 0.05 (1.73) | <MDL | <MDL | <MDL | <MDL | <MDL |
| PFHpA | 11.87 | 68.41 | 0.08 (1.63) | <MDL | <MDL | <MDL | <MDL | 0.19 |
| PFDoA | 2.21 | 58.95 | 0.28 (1.25) | <MDL | <MDL | <MDL | <MDL | <MDL |
| Et-PFOSA-AcOH | 10.66 | 70.02 | 0.02 (1.63) | <MDL | <MDL | <MDL | <MDL | 0.05 |

Abbreviations: SD, standard deviation; MDL, method detection limit. Percentile values were calculated with PFAS concentrations below the MDL coded as missing.

**Table S2. Distribution of second trimester serum levels of PFAS (ng/mL) across demographic characteristics and psychosocial stressors.**

|  | **PFNA** | | **PFOA** | | **PFOS** | | **PFHxS** | | **Me-PFOSA-AcOH** | |
| --- | --- | --- | --- | --- | --- | --- | --- | --- | --- | --- |
|  | **N** | **Geometric Mean**  **(Geometric SD)** | **N** | **Geometric Mean**  **(Geometric SD)** | **N** | **Geometric Mean**  **(Geometric SD)** | **N** | **Geometric Mean**  **(Geometric SD)** | **N** | **Geometric Mean**  **(Geometric SD)** |
| Maternal Age, years |  |  |  |  |  |  |  |  |  |  |
| 18-24 | 54 | 0.22 (1.95) | 54 | 0.57 (1.71) | 54 | 1.40 (2.05) | 54 | 0.30 (2.04) | 53 | 0.06 (2.40) |
| 25-29 | 66 | 0.21 (1.85) | 66 | 0.57 (1.83) | 66 | 1.23 (2.16) | 66 | 0.23 (2.15) | 66 | 0.05 (2.41) |
| 30-34 | 171 | 0.34 (2.08) | 172 | 0.84 (2.14) | 172 | 2.21 (2.09) | 172 | 0.45 (2.50) | 172 | 0.05 (2.01) |
| >35 | 190 | 0.32 (1.78) | 191 | 0.73 (1.89) | 191 | 1.94 (1.88) | 191 | 0.34 (1.98) | 191 | 0.05 (2.01) |
| Maternal Education |  |  |  |  |  |  |  |  |  |  |
| Less than High School | 57 | 0.22 (1.82) | 58 | 0.56 (1.82) | 58 | 1.25 (1.88) | 58 | 0.22 (1.69) | 58 | 0.05 (1.82) |
| High School Degree or Some College | 135 | 0.22 (1.87) | 135 | 0.54 (1.81) | 135 | 1.38 (2.14) | 135 | 0.25 (2.07) | 134 | 0.05 (2.40) |
| College Degree | 113 | 0.35 (1.88) | 114 | 0.85 (2.03) | 114 | 2.16 (1.98) | 114 | 0.43 (2.40) | 114 | 0.05 (2.18) |
| Graduate Degree | 181 | 0.37 (1.92) | 181 | 0.91 (1.91) | 181 | 2.44 (1.87) | 181 | 0.48 (2.13) | 181 | 0.06 (1.94) |
| Maternal Race/Ethnicity |  |  |  |  |  |  |  |  |  |  |
| White | 182 | 0.36 (1.85) | 183 | 0.94 (1.99) | 183 | 2.34 (1.81) | 183 | 0.56 (2.14) | 183 | 0.05 (1.91) |
| Black | 36 | 0.24 (1.78) | 36 | 0.50 (1.87) | 36 | 1.46 (2.09) | 36 | 0.25 (2.00) | 36 | 0.07 (1.99) |
| Asian/Pacific Islander | 82 | 0.37 (1.92) | 82 | 0.75 (1.86) | 82 | 2.30 (2.15) | 82 | 0.34 (2.08) | 82 | 0.06 (2.35) |
| Latina | 171 | 0.22 (1.85) | 172 | 0.59 (1.81) | 172 | 1.36 (2.06) | 172 | 0.24 (2.02) | 171 | 0.04 (2.12) |
| Other/Multi-Racial | 14 | 0.53 (2.57) | 14 | 1.07 (2.15) | 14 | 2.54 (1.78) | 14 | 0.43 (2.25) | 14 | 0.05 (1.65) |
| Parity |  |  |  |  |  |  |  |  |  |  |
| No Prior Births | 239 | 0.37 (1.99) | 239 | 1.02 (1.87) | 239 | 2.32 (2.02) | 239 | 0.51 (2.27) | 239 | 0.05 (2.07) |
| One or More Prior Births | 249 | 0.24 (1.83) | 251 | 0.53 (1.78) | 251 | 1.52 (2.00) | 251 | 0.25 (1.93) | 250 | 0.05 (2.15) |
| Marital Status |  |  |  |  |  |  |  |  |  |  |
| Married | 324 | 0.33 (1.96) | 326 | 0.80 (2.04) | 326 | 2.08 (2.03) | 326 | 0.41 (2.35) | 326 | 0.05 (2.12) |
| Living Together | 104 | 0.25 (2.01) | 104 | 0.62 (1.82) | 104 | 1.51 (2.10) | 104 | 0.27 (2.07) | 104 | 0.05 (1.97) |
| Single | 104 | 0.25 (1.75) | 104 | 0.61 (1.78) | 104 | 1.52 (1.99) | 104 | 0.29 (1.84) | 104 | 0.05 (2.39) |
| Perceived Stress |  |  |  |  |  |  |  |  |  |  |
| No | 421 | 0.31 (1.98) | 422 | 0.75 (2.01) | 422 | 1.90 (2.08) | 422 | 0.37 (2.31) | 421 | 0.05 (2.10) |
| Yes | 61 | 0.26 (1.86) | 62 | 0.64 (1.74) | 62 | 1.73 (2.04) | 62 | 0.31 (1.96) | 62 | 0.06 (1.90) |
| Stressful Life Events |  |  |  |  |  |  |  |  |  |  |
| No | 200 | 0.31 (1.94) | 201 | 0.78 (2.05) | 201 | 1.94 (1.98) | 201 | 0.38 (2.26) | 201 | 0.05 (1.97) |
| Yes | 283 | 0.29 (1.98) | 284 | 0.70 (1.93) | 284 | 1.82 (2.13) | 284 | 0.34 (2.27) | 283 | 0.05 (2.22) |
| Depression |  |  |  |  |  |  |  |  |  |  |
| No | 418 | 0.30 (1.99) | 420 | 0.74 (2.00) | 420 | 1.89 (2.04) | 420 | 0.37 (2.30) | 419 | 0.05 (2.11) |
| Yes | 37 | 0.28 (1.76) | 37 | 0.66 (1.84) | 37 | 1.75 (2.19) | 37 | 0.28 (1.85) | 37 | 0.07 (2.10) |
| Financial Strain |  |  |  |  |  |  |  |  |  |  |
| No | 261 | 0.37 (1.87) | 262 | 0.92 (1.94) | 262 | 2.40 (1.87) | 262 | 0.49 (2.16) | 262 | 0.05 (1.95) |
| Yes | 169 | 0.24 (1.90) | 170 | 0.57 (1.90) | 170 | 1.45 (2.12) | 170 | 0.26 (2.15) | 169 | 0.05 (2.21) |
| Food Insecurity |  |  |  |  |  |  |  |  |  |  |
| No | 408 | 0.31 (1.96) | 409 | 0.76 (1.98) | 409 | 1.96 (2.03) | 409 | 0.38 (2.28) | 408 | 0.05 (2.08) |
| Yes | 75 | 0.24 (1.95) | 76 | 0.59 (1.85) | 76 | 1.41 (2.20) | 76 | 0.24 (1.98) | 76 | 0.06 (2.32) |

Abbreviations: SD, standard deviation.

**Table S3. Crude and adjusted linear regression coefficients and 95% confidence intervals for second trimester CRH concentrations (pg/mL) in maternal plasma with an interquartile range increase in second trimester PFAS concentrations (ng/mL) in maternal serum.**

|  | **Crude^1^** | | | | **Adjusted^2^** | | |
| --- | --- | --- | --- | --- | --- | --- | --- |
|  | | **N** | **β** | **95% CI** | **N** | **β** | **95% CI** |
| PFNA | | 489 | 6.26 | (3.31, 9.21) | 464 | 5.17 | (1.79, 8.55) |
| PFOA | | 491 | 5.11 | (1.70, 8.52) | 466 | 3.62 | (-0.42, 7.66) |
| PFOS | | 491 | 4.38 | (1.06, 7.70) | 466 | 1.99 | (-1.92, 5.9) |
| PFHxS | | 490 | 0.69 | (-2.19, 3.58) | 466 | 0.33 | (-3.73, 4.39) |
| Me-PFOSA-AcOH | | 491 | 2.96 | (-0.34, 6.25) | 465 | -0.26 | (-3.28, 2.75) |

^1^Adjusted for gestational age at visit.

^2^Adjusted for gestational age at visit, maternal age (continuous), maternal education, maternal race/ethnicity, and parity.

Abbreviations: CI, confidence interval.

**Table S4. Crude and adjusted linear regression coefficients and 95% confidence intervals for the associations between psychosocial stressors and second trimester CRH concentrations (pg/mL) in maternal plasma.**

|  | **Crude^1^** | | | **Adjusted^2^** | | |
| --- | --- | --- | --- | --- | --- | --- |
|  | **N** | **β** | **95% CI** | **N** | **β** | **95% CI** |
| Financial Strain |  |  |  |  |  |  |
| No | 261 | Ref | Ref | 254 | Ref | Ref |
| Yes | 167 | -5.28 | (-11.07, 0.5) | 156 | 4.42 | (-5.42, 14.25) |
| Food Insecurity |  |  |  |  |  |  |
| No | 408 | Ref | Ref | 391 | Ref | Ref |
| Yes | 73 | -4.92 | (-12.15, 2.32) | 70 | 1.92 | (-6.25, 10.09) |
| Stressful Life Events |  |  |  |  |  |  |
| No | 201 | Ref | Ref | 197 | Ref | Ref |
| Yes | 280 | 1.66 | (-3.63, 6.95) | 264 | 3.79 | (-1.75, 9.33) |
| Perceived Stress |  |  |  |  |  |  |
| No | 419 | Ref | Ref | 404 | Ref | Ref |
| Yes | 61 | 2.57 | (-5.29, 10.43) | 57 | 10.2 | (1.82, 18.57) |
| Depression |  |  |  |  |  |  |
| No | 417 | Ref | Ref | 400 | Ref | Ref |
| Yes | 36 | -3.23 | (-13.27, 6.8) | 33 | 5.09 | (-5.67, 15.85) |

^1^Adjusted for gestational age at visit.

^2^Adjusted for gestational age at visit, maternal age (continuous), maternal education, maternal race/ethnicity, and parity.

Abbreviations: CI, confidence interval.

**Table S5. Crude and adjusted linear regression coefficients and 95% confidence intervals for second trimester CRH concentrations (pg/mL) in maternal plasma with an interquartile range increase in second trimester PFAS concentrations (ng/mL) in maternal serum stratified by stressful life events.**

|  | **Crude^1^** | | | | **Adjusted^2^** | | | |
| --- | --- | --- | --- | --- | --- | --- | --- | --- |
|  | **N** | **β** | **95% CI** | **p interaction** | **N** | **β** | **95% CI** | **p interaction** |
| PFNA |  |  |  |  |  |  |  |  |
| Stressful Life Events– Yes | 279 | 7.62 | (3.95, 11.29) | 0.53 | 263 | 7.06 | (2.82, 11.3) | 0.49 |
| Stressful Life Events– No | 200 | 5.59 | (0.57, 10.61) |  | 196 | 2.29 | (-3.45, 8.03) |  |
| PFOA |  |  |  |  |  |  |  |  |
| Stressful Life Events– Yes | 280 | 4.84 | (0.43, 9.25) | 0.9 | 264 | 3.62 | (-1.76, 8.99) | 0.40 |
| Stressful Life Events– No | 201 | 5.24 | (0.00, 10.48) |  | 197 | 3.56 | (-2.86, 9.98) |  |
| PFOS |  |  |  |  |  |  |  |  |
| Stressful Life Events– Yes | 280 | 5.26 | (1.03, 9.50) | 0.71 | 264 | 2.77 | (-2.10, 7.65) | 0.79 |
| Stressful Life Events– No | 201 | 6.43 | (0.46, 12.4) |  | 197 | 1.00 | (-5.90, 7.91) |  |
| PFHxS |  |  |  |  |  |  |  |  |
| Stressful Life Events– Yes | 280 | 3.63 | (-0.58, 7.84) | 0.98 | 264 | 0.86 | (-4.33, 6.06) | 0.89 |
| Stressful Life Events– No | 201 | 3.59 | (-1.88, 9.07) |  | 197 | -0.42 | (-7.16, 6.32) |  |
| Me-PFOSA-AcOH |  |  |  |  |  |  |  |  |
| Stressful Life Events– Yes | 279 | 0.24 | (-3.20, 3.69) | 0.69 | 263 | -0.89 | (-4.66, 2.88) | 0.93 |
| Stressful Life Events– No | 201 | 1.5 | (-3.74, 6.74) |  | 197 | 0.65 | (-4.55, 5.85) |  |

^1^Adjusted for gestational age at visit.

^2^Adjusted for gestational age at visit, maternal age (continuous), maternal education, maternal race/ethnicity, and parity.

Abbreviations: CI, confidence interval.

**Table S6. Crude and adjusted linear regression coefficients and 95% confidence intervals for second trimester CRH concentrations (pg/mL) in maternal plasma with an interquartile range increase in second trimester PFAS concentrations (ng/mL) in maternal serum stratified by perceived stress.**

|  | **Crude^1^** | | | | **Adjusted^2^** | | | |
| --- | --- | --- | --- | --- | --- | --- | --- | --- |
|  | **N** | **β** | **95% CI** | **p interaction** | **N** | **β** | **95% CI** | **p interaction** |
| PFNA |  |  |  |  |  |  |  |  |
| Perceived Stress – Yes | 60 | 3.87 | (-5.04, 12.78) | 0.5 | 56 | 3.45 | (-6.59, 13.48) | 0.55 |
| Perceived Stress – No | 418 | 7.23 | (4.05, 10.41) |  | 403 | 5.55 | (1.92, 9.19) |  |
| PFOA |  |  |  |  |  |  |  |  |
| Perceived Stress – Yes | 61 | -1.00 | (-12.08, 10.09) | 0.29 | 57 | -3.15 | (-15.91, 9.60) | 0.44 |
| Perceived Stress – No | 419 | 5.59 | (2.03, 9.14) |  | 404 | 4.61 | (0.28, 8.95) |  |
| PFOS |  |  |  |  |  |  |  |  |
| Perceived Stress – Yes | 61 | 4.25 | (-5.40, 13.9) | 0.8 | 57 | 4.76 | (-6.32, 15.84) | 0.86 |
| Perceived Stress – No | 419 | 5.63 | (1.91, 9.35) |  | 404 | 1.69 | (-2.58, 5.95) |  |
| PFHxS |  |  |  |  |  |  |  |  |
| Perceived Stress – Yes | 61 | 2.70 | (-8.14, 13.55) | 0.88 | 57 | 2.80 | (-8.91, 14.51) | 0.97 |
| Perceived Stress – No | 419 | 3.66 | (0.12, 7.2) |  | 404 | 0.41 | (-4.04, 4.86) |  |
| Me-PFOSA-AcOH |  |  |  |  |  |  |  |  |
| Perceived Stress – Yes | 61 | -2.82 | (-11.84, 6.21) | 0.47 | 57 | -3.66 | (-13.61, 6.92) | 0.99 |
| Perceived Stress – No | 418 | 0.97 | (-2.22, 4.15) |  | 403 | -0.43 | (-3.68, 2.81) |  |

^1^Adjusted for gestational age at visit.

^2^Adjusted for gestational age at visit, maternal age (continuous), maternal education, maternal race/ethnicity, and parity.

Abbreviations: CI, confidence interval.

Note: among those who experienced perceived stress, no participants self-identified as “other/multi-racial” race/ethnicity and maternal race/ethnicity was modeled as 4 categories.

**Table S7. Crude and adjusted linear regression coefficients and 95% confidence intervals for second trimester CRH concentrations (pg/mL) in maternal plasma with an interquartile range increase in second trimester PFAS concentrations (ng/mL) in maternal serum stratified by depression.**

|  | **Crude^1^** | | | | **Adjusted^2^** | | | |
| --- | --- | --- | --- | --- | --- | --- | --- | --- |
|  | **N** | **β** | **95% CI** | **p interaction** | **N** | **β** | **95% CI** | **p interaction** |
| PFNA |  |  |  |  |  |  |  |  |
| Depression – Yes | 36 | 13.21 | (2.03, 24.39) | 0.33 | 33 | 20.77 | (1.56, 39.99) | 0.47 |
| Depression – No | 415 | 6.41 | (3.19, 9.64) |  | 398 | 4.9 | (1.23, 8.56) |  |
| PFOA |  |  |  |  |  |  |  |  |
| Depression – Yes | 36 | 4.65 | (-7.83, 17.14) | 0.99 | 33 | 7.06 | (-11.96, 26.07) | 0.4 |
| Depression – No | 417 | 4.78 | (1.15, 8.41) |  | 400 | 3.78 | (-0.65, 8.21) |  |
| PFOS |  |  |  |  |  |  |  |  |
| Depression – Yes | 36 | 1.16 | (-10.68, 13.0) | 0.45 | 33 | -3.88 | (-27.33, 19.56) | 0.74 |
| Depression – No | 417 | 6.19 | (2.32, 10.06) |  | 400 | 2.73 | (-1.71, 7.17) |  |
| PFHxS |  |  |  |  |  |  |  |  |
| Depression – Yes | 36 | -0.87 | (-15.86, 14.12) | 0.58 | 33 | -10.23 | (-41.98, 21.52) | 0.83 |
| Depression – No | 417 | 3.91 | (0.3, 7.52) |  | 400 | 1.16 | (-3.33, 5.64) |  |
| Me-PFOSA-AcOH |  |  |  |  |  |  |  |  |
| Depression – Yes | 36 | -4.45 | (-14.31, 5.41) | 0.3 | 33 | -10.12 | (-29.15, 8.91) | 0.93 |
| Depression – No | 416 | 1.68 | (-1.53, 4.88) |  | 399 | 0.37 | (-2.92, 3.66) |  |

^1^Adjusted for gestational age at visit.

^2^Adjusted for gestational age at visit, maternal age (continuous), maternal education, maternal race/ethnicity, and parity.

Abbreviations: CI, confidence interval.

**Table S8. Crude and adjusted linear regression coefficients and 95% confidence intervals for second trimester CRH concentrations (pg/mL) in maternal plasma with an interquartile range increase in second trimester PFAS concentrations (ng/mL) in maternal serum stratified by food insecurity.**

|  | **Crude^1^** | | | | **Adjusted^2^** | | | |
| --- | --- | --- | --- | --- | --- | --- | --- | --- |
|  | **N** | **β** | **95% CI** | **p interaction** | **N** | **β** | **95% CI** | **p interaction** |
| PFNA |  |  |  |  |  |  |  |  |
| Food Insecurity – Yes | 72 | 8.41 | (1.51, 15.3) | 0.62 | 69 | 9.52 | (1.72, 17.32) | 0.56 |
| Food Insecurity – No | 407 | 6.13 | (2.83, 9.42) |  | 390 | 4.68 | (0.89, 8.48) |  |
| PFOA |  |  |  |  |  |  |  |  |
| Food Insecurity – Yes | 73 | 6.6 | (-1.89, 15.09) | 0.76 | 70 | 5.06 | (-4.91, 15.03) | 0.46 |
| Food Insecurity – No | 408 | 4.48 | (0.78, 8.19) |  | 391 | 3.75 | (-0.79, 8.29) |  |
| PFOS |  |  |  |  |  |  |  |  |
| Food Insecurity – Yes | 73 | 5.51 | (-1.97, 12.99) | 0.99 | 70 | 8.07 | (-0.92, 17.06) | 0.83 |
| Food Insecurity – No | 408 | 5.23 | (1.31, 9.15) |  | 391 | 1.78 | (-2.71, 6.26) |  |
| PFHxS |  |  |  |  |  |  |  |  |
| Food Insecurity – Yes | 73 | 8.2 | (-0.9, 17.3) | 0.31 | 70 | 8.2 | (-3.12, 19.51) | 0.96 |
| Food Insecurity – No | 408 | 2.42 | (-1.25, 6.09) |  | 391 | -0.11 | (-4.55, 4.33) |  |
| Me-PFOSA-AcOH |  |  |  |  |  |  |  |  |
| Food Insecurity – Yes | 73 | 0.32 | (-5.62, 6.26) | 0.91 | 70 | 2.99 | (-3.79, 9.78) | 0.99 |
| Food Insecurity – No | 407 | 0.95 | (-2.34, 4.25) |  | 390 | -0.56 | (-4.09, 2.97) |  |

^1^Adjusted for gestational age at visit.

^2^Adjusted for gestational age at visit, maternal age (continuous), maternal education, maternal race/ethnicity, and parity.

Abbreviations: CI, confidence interval.

**Table S9. Crude and adjusted linear regression coefficients and 95% confidence intervals for second trimester CRH concentrations (pg/mL) in maternal plasma with an interquartile range increase in second trimester PFAS concentrations (ng/mL) in maternal serum stratified by financial strain.**

|  | **Crude^1^** | | | | **Adjusted^2^** | | | |
| --- | --- | --- | --- | --- | --- | --- | --- | --- |
|  | **N** | **β** | **95% CI** | **p interaction** | **N** | **β** | **95% CI** | **p interaction** |
| PFNA |  |  |  |  |  |  |  |  |
| Financial Strain – Yes | 166 | 11.56 | (6.56, 16.55) | 0.02 | 155 | 10.61 | (4.9, 16.33) | 0.79 |
| Financial Strain – No | 260 | 2.57 | (-2.13, 7.26) |  | 253 | 3.00 | (-2.05, 8.05) |  |
| PFOA |  |  |  |  |  |  |  |  |
| Financial Strain – Yes | 167 | 8.16 | (2.33, 13.98) | 0.12 | 156 | 6.1 | (-0.49, 12.69) | 0.68 |
| Financial Strain – No | 261 | 1.22 | (-3.79, 6.22) |  | 254 | 3.03 | (-3.09, 9.15) |  |
| PFOS |  |  |  |  |  |  |  |  |
| Financial Strain – Yes | 167 | 8 | (2.48, 13.53) | 0.11 | 156 | 4.63 | (-1.82, 11.09) | 0.99 |
| Financial Strain – No | 261 | 0.2 | (-5.63, 6.03) |  | 254 | -0.05 | (-6.09, 5.99) |  |
| PFHxS |  |  |  |  |  |  |  |  |
| Financial Strain – Yes | 167 | 5.24 | (-0.59, 11.08) | 0.09 | 156 | 1.59 | (-5.01, 8.18) | 0.81 |
| Financial Strain – No | 261 | -1.55 | (-6.65, 3.55) |  | 254 | -1.01 | (-7.01, 4.99) |  |
| Me-PFOSA-AcOH |  |  |  |  |  |  |  |  |
| Financial Strain – Yes | 166 | 1.41 | (-3.11, 5.93) | 0.66 | 155 | 0.23 | (-4.84, 5.3) | 0.87 |
| Financial Strain – No | 261 | -0.34 | (-5.02, 4.35) |  | 254 | -0.02 | (-5.01, 4.98) |  |

^1^Adjusted for gestational age at visit.

^2^Adjusted for gestational age at visit, maternal age (continuous), maternal education, maternal race/ethnicity, and parity.

Abbreviations: CI, confidence interval.
